# Cellular and molecular mechanisms of pathological tau phosphorylation in traumatic brain injury: implications for chronic traumatic encephalopathy

**DOI:** 10.1186/s13024-025-00842-z

**Published:** 2025-05-10

**Authors:** Neil Donison, Jacqueline Palik, Kathryn Volkening, Michael J. Strong

**Affiliations:** 1 Molecular Medicine Group, Robarts Research Institute, Western University, London, ON Canada; 2 Neuroscience Graduate Program, Schulich School of Medicine and Dentistry, Western University, London, ON Canada; 3 Clinical Neurological Sciences, Schulich School of Medicine and Dentistry, Western University, London, ON Canada; 4 Pathology and Laboratory Medicine, Schulich School of Medicine and Dentistry, Western University, London, ON Canada

**Keywords:** Tau, Tauopathy, Chronic traumatic encephalopathy, Traumatic brain injury, Phosphorylation, Neurodegeneration, Neuroinflammation, Excitotoxicity, Mitochondrial dysfunction, Oxidative stress

## Abstract

Tau protein plays a critical role in the physiological functioning of the central nervous system by providing structural integrity to the cytoskeletal architecture of neurons and glia through microtubule assembly and stabilization. Under certain pathological conditions, tau is aberrantly phosphorylated and aggregates into neurotoxic fibrillary tangles. The aggregation and cell-to-cell propagation of pathological tau leads to the progressive deterioration of the nervous system. The clinical entity of traumatic brain injury (TBI) ranges from mild to severe and can promote tau aggregation by inducing cellular mechanisms and signalling pathways that increase tau phosphorylation and aggregation. Chronic traumatic encephalopathy (CTE), which is a consequence of repetitive TBI, is a unique tauopathy characterized by pathological tau aggregates located at the depths of the sulci and surrounding blood vessels. The mechanisms leading to increased tau phosphorylation and aggregation in CTE remain to be fully defined but are likely the result of the primary and secondary injury sequelae associated with TBI. The primary injury includes physical and mechanical damage resulting from the head impact and accompanying forces that cause blood–brain barrier disruption and axonal shearing, which primes the central nervous system to be more vulnerable to the subsequent secondary injury mechanisms. A complex interplay of neuroinflammation, oxidative stress, excitotoxicity, and mitochondrial dysfunction activate kinase and cell death pathways, increasing tau phosphorylation, aggregation and neurodegeneration. In this review, we explore the most recent insights into the mechanisms of tau phosphorylation associated with TBI and propose how multiple cellular pathways converge on tau phosphorylation, which may contribute to CTE progression.

**Graphical Abstract:**

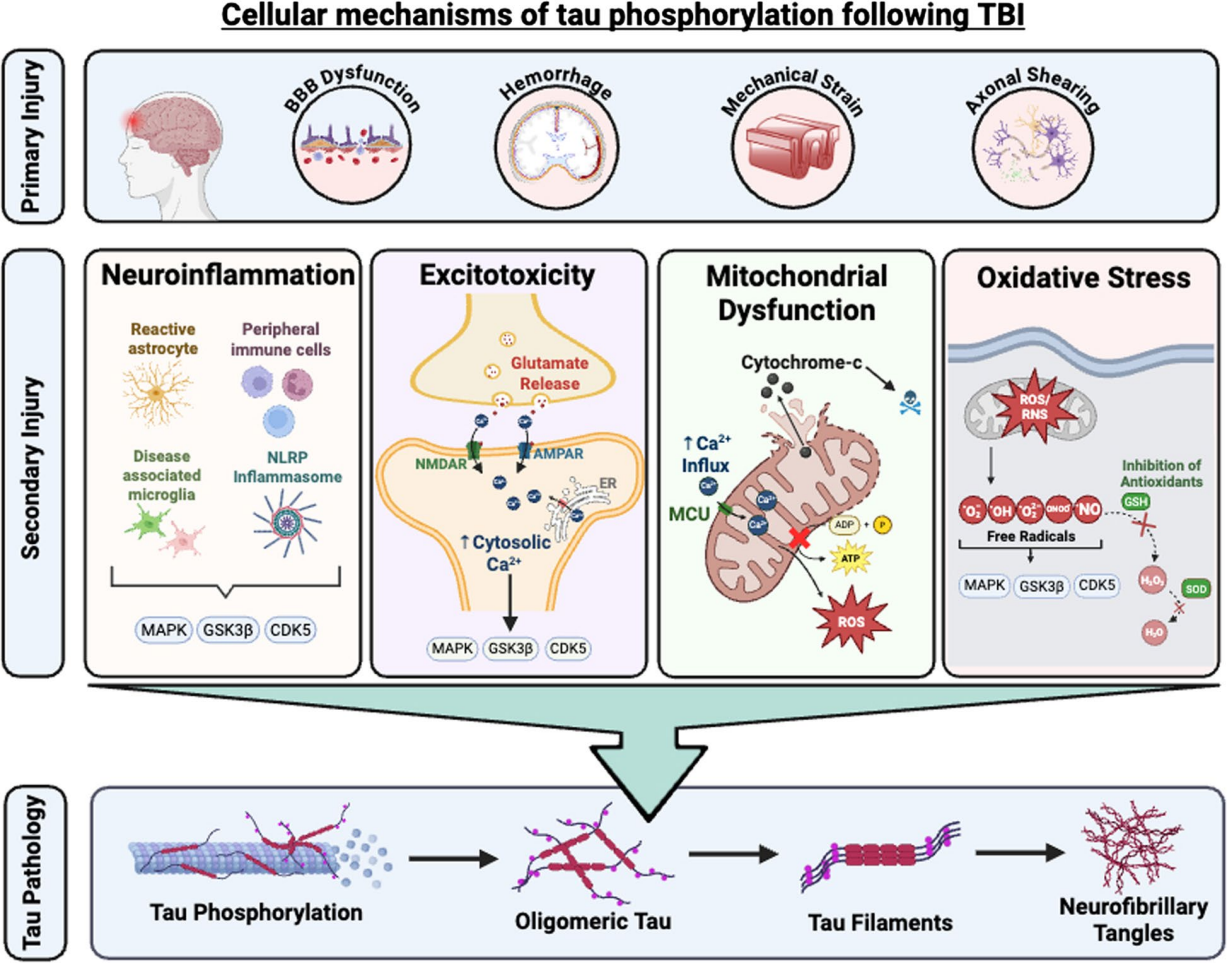

## Background

Traumatic brain injury (TBI) is one of the leading causes of disability and has been associated with an increased prevalence of dementia [1]. The complex nature of TBI is characterized by a diverse pathophysiological response. Chronic activation of the cellular and molecular sequelae following TBI is linked to increased dysfunction and aggregation of various proteins, including tau [2, 3], amyloid beta (Aβ) [4, 5], alpha-synuclein [6, 7] and TAR DNA-binding protein 43 (TDP-43) [8, 9]. TBI can range in severity on a spectrum from mild to severe. Mild TBI (mTBI), often referred to as a concussion, is the most common type of TBI, and typically presents with clinical symptoms such as confusion or disorientation, dizziness, amnesia, depression and sleep disturbances that resolve spontaneously within days to weeks [10, 11]. More recently, subconcussive TBIs (sometimes categorized as mTBI), which do not result in any noticeable symptoms, have also been suggested to lead to neurological alterations, particularly when repetitive [12]. Although it has been suggested that TBI can increase the risk for various neurodegenerative diseases, including Alzheimer’s disease [13–15], Parkinson’s disease [16], and amyotrophic lateral sclerosis (ALS) [17], the neuropathological basis of this remains to be fully defined. More recently it has been shown that exposure to repeated TBI or head impacts, both concussive and subconcussive, is the primary risk factor for chronic traumatic encephalopathy (CTE) which is a unique neurodegenerative disease characterized by neuropathologically distinct inclusions of filamentous phosphorylated tau protein [18, 19]. Given that CTE is defined by both exposure to repetitive TBI and the presence of phosphorylated tau aggregates, understanding the link between the two is essential. Despite this, the exact molecular mechanisms that lead to tau phosphorylation and aggregation as a result of TBI are complex and not fully understood.

In this review we examine the cellular and molecular mechanisms associated with TBI and how they contribute to tau phosphorylation. We primarily focus on the role that neuroinflammation, excitotoxicity, mitochondrial dysfunction and oxidative stress play in the phosphorylation and aggregation of tau through the activation of various kinases and cellular mediators. While past reviews have examined the various injury responses associated with TBI from a broad perspective, we solely investigated how these mechanisms contribute to tau phosphorylation. Moreover, reviews on the cellular mechanisms of tau phosphorylation in TBI and CTE are limited but critical, given our increasing knowledge of the heterogeneity between tauopathies. The purpose of this review is to provide insights into how mechanisms of TBI-induced tau phosphorylation may contribute to the development of CTE. We compiled clinical and basic research data from various fields to provide the most up-to-date scoping review on tau phosphorylation in TBI and CTE. We propose a detailed perspective on the comprehensive cellular network that contributes to tau phosphorylation in the context of TBI, with implications for CTE. In doing so, we acknowledge that certain associations and inferences were made between clinical and basic research that have yet to be validated in humans. In addition, we also review the neuropathology of CTE, briefly outline important tau-kinases and discuss strain variability between CTE and other tauopathies.

### Chronic traumatic encephalopathy

The first reports of what we now call CTE were recorded in the early twentieth century by Harrison Martland in boxers who had been exposed to repetitive neurotrauma [20]. Martland described boxers as having ‘Punch-Drunk’ syndrome, evidenced by symptoms of cognitive dysfunction and Parkinsonian-like motor deficits, which was later defined as dementia pugilistica [21]. Although the first neuropathological correlates of dementia pugilistica were observed as cortical neurodegeneration and abnormal tau protein deposition in a subset of 15 boxers [22], the effects of repeated TBI on brain health largely remained understudied until the early twenty-first century when CTE was re-introduced into the scientific literature. In 2005, Omalu et al. described the presence of tau pathology in two American football players who had displayed severe clinical symptoms prior to death [23]. This was followed by the diagnosis of CTE in other contact sport athletes and military veterans over the next two decades [8, 24–28]. Of note, the histopathology of CTE differs from Alzheimer’s disease and other tauopathies, which supports the causative role of repeated TBI and head impacts in the development and progression of this disorder. To date, CTE has been diagnosed in over 400 athletes across multiple sports, with recent findings of the first cases of CTE in a female athlete [29] and young adults [18].

Over the past decade, our knowledge and understanding of the long-term consequences of repeated head impacts and TBI as a risk factor for neurodegenerative diseases has increased such that it has led to global public attention. Currently, CTE can only be confirmed post-mortem by neuropathological examination. As outlined in the most recent National Institutes of Neurological Disorders and Stroke (NINDS) and National Institute of Biomedical Imaging and Bioengineering (NBIB) consensus pathological criteria, CTE is defined by the unique presence of abnormally phosphorylated tau (p-tau) inclusions in neurons and astrocytes at the depths of the sulci and surrounding blood vessels [19]. p-Tau lesions in the superficial layers II-III, CA2 and CA4 hippocampal subregions, and other subcortical nuclei, are additional features that support the pathological diagnosis of CTE.

Given that CTE is exclusively a neuropathological-based diagnosis, there is an increasing urgency to better understand the disease in living patients. In addition to the neuropathological features of CTE, specific clinical symptoms have also been described in CTE patients based on retrospective recollections and analysis [30–32]. Impairments in cognitive domains such as attention, episodic memory, and executive function, including florid dementia, are the most highly associated clinical features of pathological CTE [30, 33]. Other clinical features include behavioural alterations such as violence, impulsivity, aggression and rage; psychiatric disorders, including depression, anxiety and apathy; and motor problems, including disturbances in gait and balance, dysarthria and motor control. Recently, these symptoms have been used as part of the diagnostic criteria for traumatic encephalopathy syndrome (TES), a clinical disorder associated with the neuropathological correlates of CTE [30]. The NINDS Consensus Diagnostic Criteria for TES consists of a four-step process in which all of the first three parameters must be met to confirm a diagnosis of TES, followed by an assessment for functional dependence, which ranges from independent to severe dementia. The first three criteria include: 1) exposure to repeated head impacts, 2) core clinical features, including cognitive impairment and neurobehavioural dysregulation, and 3) the symptoms must not be accounted for by other disorders (although a comorbid diagnosis does not fully exclude TES). Furthermore, the NINDS also provided criteria for Provisional Levels of Certainty for CTE based on features including delayed onset, motor signs and psychiatric features. The four levels of certainty are 1) Suggestive, 2) Possible, 3) Probable, and 4) Definitive CTE with TES (confirmed post-mortem). While it is important to note that the diagnosis of TES is not intended as a diagnosis of CTE, it is a critical component to fill the gaps in knowledge that are inherently present in patient and caregiver reporting, which can be subjective and non-descriptive. More importantly, this will allow for longitudinal studies to compare the association between TES and neuropathologically diagnosed CTE.

Immense strides have been taken to better understand the relationship between TBI and CTE. As a result, the development of diagnostic biomarkers and therapeutic treatments to prevent CTE and other tauopathies has been prioritized. Much of the focus has centred around the pathophysiology of tau as it is the key neuropathological hallmark. Imaging and fluid biomarkers have been developed to detect the changes in tau protein prior to the development of symptoms and irreversible damage [34, 35]. Therapeutic strategies aim to target or reduce tau post-translational modifications, aggregation, or expression (reviewed in [36]). While these have shown promise, by and large, the majority have yet to succeed in clinical trials.

### Tau protein

#### Tau structure and function

**Fig. 1:**
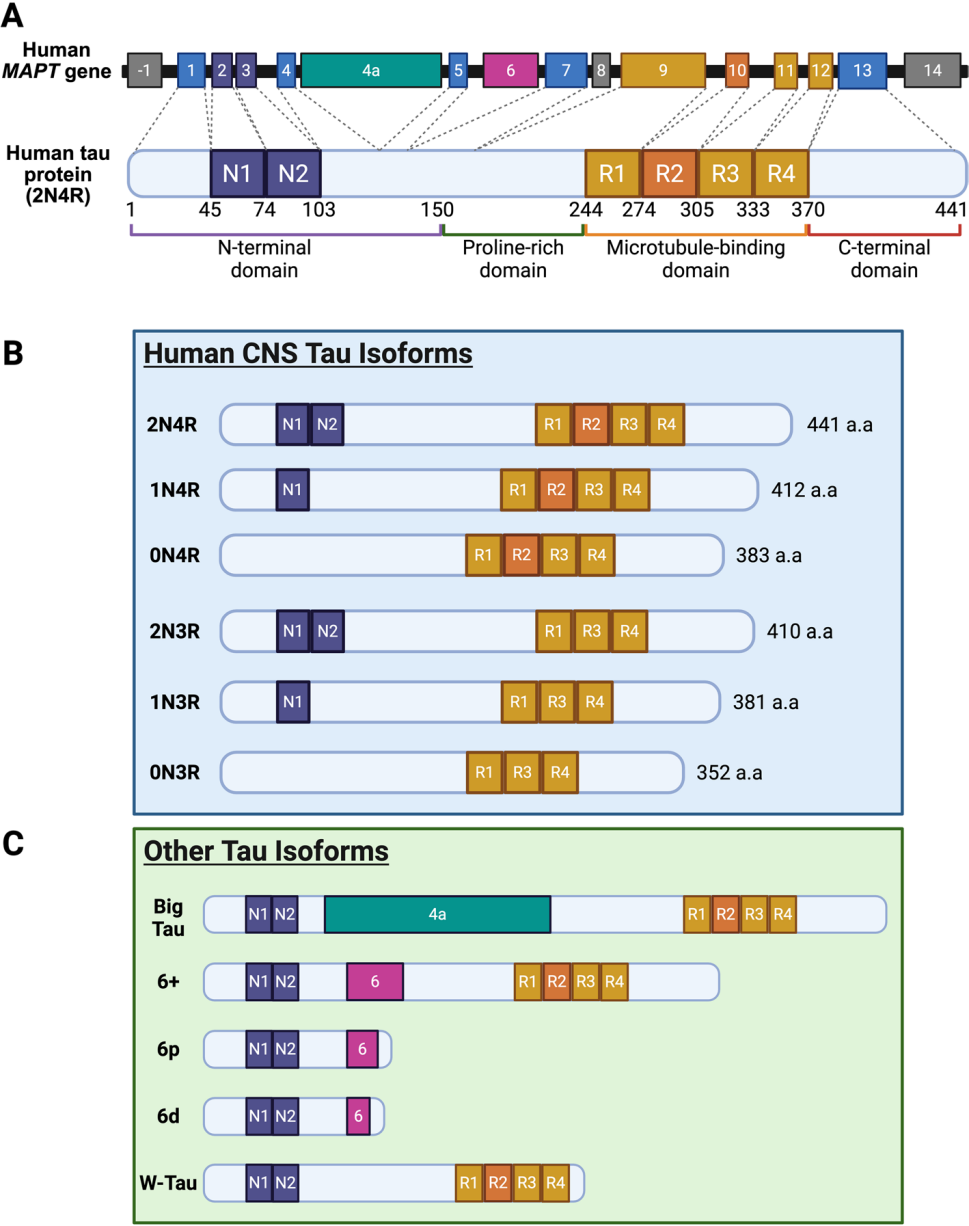
Tau protein structure and isoforms. **a** Tau protein is encoded by the *MAPT* gene located on chromosome 17, consisting of 16 exons. Exons 1, 4, 5, 7, 9, 11, 12 and 13 are constitutively expressed in all human tau isoforms. Exons −1, 8 and 14 are never transcribed, while exons 4a and 6 are only transcribed in non-canonical isoforms. Exons 2 and 3 encode for the N-terminal repeats, N1 and N2, respectively. Exons 9–12 encode four repeat motifs, R1-R4, which comprise the microtubule-binding domain (MTBD). In general, tau consists of four domains, the N-terminus, Proline-rich domain, MTBD and the C-terminus. **b** Alternative splicing exons 2, 3 and 10 produces six unique human isoforms, characterized by the inclusion and exclusion of N1, N2 and R2. Isoforms range in size from 352 amino acids (0 N3R) to 441 amino acids (2 N4R). In the healthy adult human CNS, 3R and 4R isoforms are expressed in relatively equal ratios, and disruption of this balance is implicated in tauopathies. **c** Additional tau isoforms have been described in humans and other species. The inclusion of exon 4a produces a large isoform termed Big Tau. Other isoforms include the presence of exon 6 (6 +, 6p and 6 d tau) or a truncated isoform that includes a portion of intron 12 (W-tau). Created in BioRender

The microtubule-associated protein tau is a multifunctional protein highly expressed in the central and peripheral nervous system. Tau protein is primarily enriched in the axons of neurons [37] and, to a lesser extent, in glial cells [38, 39]. Tau is encoded by the *MAPT* gene, located on chromosome 17 at the band position q21.31 in humans [40]. The human *MAPT* gene contains 16 exons, of which exons −1 and 14 are never translated, while exon 8 inclusion has only been described in bovines (Fig. 1A). Alternative splicing of exons 2, 3 and 10 of *MAPT* results in six unique isoforms of tau that are found in the human central nervous system (CNS), which contain either zero, one, or two N-terminal repeats and three or four microtubule-binding repeats [41, 42] (Fig. 1B). The longest isoform, 2 N4R tau, is 441 amino acids (aa) in length, while the shortest isoform, 0 N3R, is 352 aa. In the adult human brain, there is a relatively equal ratio of 3R and 4R tau [41, 43] which is critical to healthy nervous system functioning as evidenced by an apparent change in the ratio in certain disease conditions [44–46]. For example, Pick’s disease is a primary tauopathy with a predominant 3R tau expression while progressive supranuclear palsy (PSP) and corticobasal degeneration (CBD) express increased 4R tau. Some tauopathies, such as CTE and Alzheimer’s disease, are considered mixed tauopathies where neuropathological inclusions contain both 3R and 4R tau species. In addition to the six canonical CNS tau isoforms, other isoforms have been described in various species (Fig. 1C). Most notably, a 110 kDa large variant of the 2 N4R isoform, known as ‘big tau’, which includes the 250 aa spanning exon 4a, has been reported mainly in the adult peripheral nervous system and in select regions of the CNS [47, 48]. Despite its discovery in the early 1990 s, its role in health and disease is not well understood (reviewed in [49]). Other isoforms of tau that have been described include a set of three isoforms which contain exon 6, termed 6 +, 6p and 6 d [50–52], and W-tau, which is a truncated isoform that occurs because of the retention of intron 12 [53, 54].

In general, tau is composed of four unique domains which play a critical role in its physiology and function. The longest isoform, 2 N4R tau, contains a N-terminal domain (aa 1–150) that includes the N-terminal repeats (aa 45–103) and a short motif of 17 amino acids (aa 2–19) known as the phosphatase activating domain (PAD). The PAD plays an essential role in regulating fast axonal transport [55] and exposure of this domain in disease disrupts this process [56, 57]. Moreover, PAD exposure is also critical in the induction of subsequent tau aggregation via kinase activation and tau phosphorylation [58]. Downstream of the N-terminal domain is the proline-rich domain (aa 151–243) which contains seven Pro-X-X-Pro motifs known to interact with various proteins, including kinases such as Fyn and Src, phosphatases, peptidyl-prolyl cis/trans isomerase NIMA-interacting 1 (Pin1) and Bridging integrator 1 [59]. Amino acids 244–369 contain four imperfect repeated motifs constituting the microtubule-binding domain (MTBD). The MTBD plays a critical role in the binding of tau to microtubules for assembly and stability [60–63] and also in tau aggregation and fibril formation where the MTBD constitutes the amyloidogenic core of neurofibrillary tangles (NFTs) [64, 65]. The fourth domain of tau is the C-terminus (aa 370–441) which impacts microtubule stability and tau toxicity [66, 67].

Tau is an intrinsically disordered protein with high conformational flexibility [68, 69] and thus its exact structure has been challenging to study. Tau is believed to be natively unfolded but has been demonstrated to adopt a ‘paperclip’ conformation when in solution in which the N-terminal projection domain folds over and interacts with the C-terminal region [70]. Loss of this conformation is considered critical in the aggregation of tau. Further, when tau is bound to microtubules it has a unique conformation such that the MTBD interacts with the tubulin subunits, while the N-terminal extends into the cytoplasm [60, 64, 71]. The intrinsically disordered properties of tau give rise to its diverse set of functions.

Tau was initially identified as a microtubule associated protein [72] where it was demonstrated to play an important role in promoting the assembly and stabilization of microtubules in vitro [73]. Soon thereafter, tau was discovered to be the main component of NFTs in Alzheimer's disease [74–77]. Tau also plays a role in various other physiological functions, such as regulating fast axonal transport by modulating the activity of kinesin and dynein [55, 78–80]. The N-terminal PAD can directly interact with and activate protein phosphatase-1, which in turn activates glycogen synthase kinase 3 beta (GSK3β) and subsequently phosphorylates the light chain of kinesin-1, disrupting its ability to bind to cargo [57, 78]. Other studies have demonstrated that tau can directly interfere with the binding of kinesin and dynein to microtubules [81, 82]. In addition to regulating microtubule physiology in neurons, tau can also localize in other cellular compartments including dendrites [83], growth cones [84, 85], the plasma membrane [86], and the nucleus [87, 88]. Nuclear tau is proposed to contribute to the maintenance of DNA and RNA integrity upon cellular stress, regulate transcription, and stabilize chromosomes [89]. Understanding the function of tau in physiological conditions is still in its relative infancy, but it has been critical to better understand its role in neurodegenerative diseases.

#### MAPT mutations and genetic modifiers

To date, over 50 pathogenic mutations have been identified in the *MAPT* gene [90]. Pathogenic mutations in *MAPT* have been shown to cause some forms of frontotemporal dementia (FTD), a group of diseases characterized by deficits in behaviour, language or movement [91]. In approximately 30–40% of FTD cases, frontal and temporal lobe atrophy is accompanied by phosphorylated tau inclusions, termed frontotemporal lobar degeneration (FTLD) with tau. Frontotemporal dementia with parkinsonism linked to chromosome 17 (FTDP-17) was the first FTLD-tau syndrome identified that was causally linked to autosomal dominant mutations in *MAPT* [44, 92–94]. This discovery provided direct evidence for the first time that tau dysfunction and aggregation itself is capable of driving neurodegeneration and clinical symptomology. Since then, more mutations in *MAPT* have been identified and linked to other primary tauopathies such as Pick’s disease, PSP and CBD, including R5L, K257 T, P301L, P301S, S305 N, V337M, and R406 W [90]. Other mutations in *MAPT* have been associated with an increased risk of tauopathy, such as the A152 T variant for Alzheimer’s disease [95].

In general, most *MAPT* mutations result in increased aggregation of phosphorylated tau and neurodegeneration regardless of the mechanisms by which the mutations may disrupt tau biology, including alterations in microtubule binding, changes in alternative splicing or increased propensity to aggregate or seed [90]. *MAPT* mutations are either intronic or exonic and are typically grouped into three categories. The majority of mutations are missense mutations in exon 10, which result in alterations in the second MTBD repeat that is specific to 4R tau isoforms. Mutations in the intronic stem-loop structure adjacent to exon 10 result in alterations of splicing, leading to increased inclusion or exclusion of exon 10 and subsequent shifts in the 3R:4R ratio. Lastly, there is a group of missense mutations located outside of the MTBD that affect all isoforms of tau and generally result in increased tau aggregation and reduced microtubule assembly.

To date, there is no evidence that has linked specific *MAPT* mutations to CTE, but it is likely that some mutations may increase risk susceptibility or lower the age of disease onset. A study of 17 CTE cases demonstrated a non-significant trend towards an increased frequency of homozygous *MAPT* H1/H1 haplotype in CTE patients compared to those without CTE [96]. Other genetic risk factors, including apolipoprotein E ε4 (APOE4) [97] and variations in transmembrane protein 106B (TMEM106B) [98], are associated with CTE and have been suggested to affect tau pathology and CTE indirectly. Patients harbouring the APOE4 allele had a 2.34-fold greater risk of developing more severe CTE and worsened p-tau burden [97]. It is proposed that these mutations impart their effects by contributing to the widespread neuroinflammatory response associated with TBI and CTE. Overall, a greater understanding of the role that genetics and *MAPT* mutations play in CTE may provide some context as to why some patients exposed to repeated TBI are more susceptible to CTE compared to others.

#### Tau post-translational modifications

Tau is subject to several different types of post-translational modifications, including phosphorylation, truncation, isomerization, acetylation, methylation, N-glycosylation, O-GlcNAcylation, glycation, nitration, oxidation, SUMOylation and ubiquitination [99]. Tau phosphorylation is the most widely studied post-translational modification due to its impact on aggregation and pathological fibril formation. Although the importance of the other post-translational modifications on tau physiology remains to be fully elucidated, they have been shown to contribute to changes in tau function and localization [100–104] and thus represent possible avenues for future diagnostic or therapeutic targets.

#### Phosphorylation

**Fig. 2:**
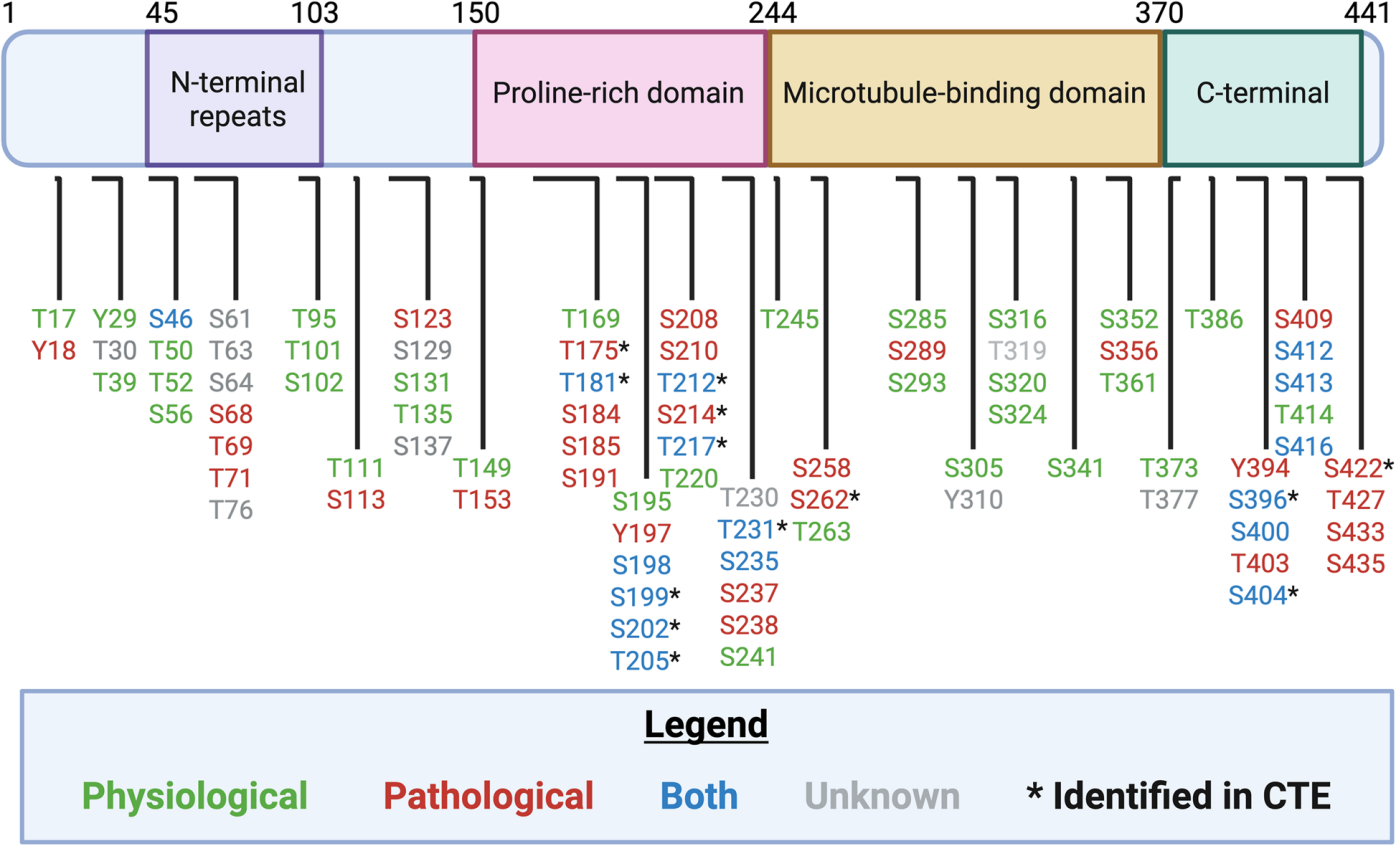
Tau phosphorylation sites. Tau protein consists of 85 potential phosphorylation sites, some of which contribute to the physiological function of tau (green), while others have been implicated in the pathogenesis of tau (red). Certain sites have been described as contributing to both the physiological and pathological roles of tau (blue), while other phosphorylation sites have yet to be demonstrated in humans or have an unknown effect on the function of tau (grey). In CTE, consensus phosphorylation sites have been demonstrated to be present in post-mortem analysis and are implicated in driving tau pathogenicity (black asterisk). Created in BioRender

The discovery that tau can promote neurodegeneration is linked to its characterization in Alzheimer’s disease patients in which hyperphosphorylated tau is the primary component of intracellular filamentous aggregates known as NFTs [74, 76, 77]. The pathogenicity of tau was further supported by the identification of mutations in the *MAPT* gene that can cause autosomal dominantly inherited FTLDs, characterized by the presence of phosphorylated neuronal and glial tau pathology [44, 93, 94]. Many studies have since investigated the role of tau phosphorylation in neurodegenerative disease. With respect to 2 N4R tau, there are 85 residues capable of undergoing phosphorylation, including 45 serine residues, 35 threonine residues and five tyrosine residues, while this number is likely closer to 50 putative sites in neurodegenerative disease [105]. Given the extensive characterization and mapping of phosphorylation sites in disease and health, specific sites have been identified as physiological such that they are found to be phosphorylated in non-diseased states and throughout development, where they promote microtubule assembly and reduce aggregation. In contrast, others are classified as pathological based on their increased presence in disease and ability to drive pathogenesis (Fig. 2).

Tau phosphorylation is present in the CNS under both physiological and pathological conditions, highlighting the importance of phosphorylation in normal tau function. However, this is a tightly regulated process involving kinases and phosphatases that are sensitive to specific cellular conditions. A disruption in the balance of phosphorylation due to changes in kinase and phosphatase activity can lead to abnormal levels of tau phosphorylation and, thus, more prone to a pathological state. During neurodevelopment, 0 N3R tau is predominantly expressed in the fetal brain (hence referred to as fetal tau) [41] and is highly phosphorylated compared to tau in the adult brain [106–109]. Fetal tau has been shown to be phosphorylated at sites that are also phosphorylated in tauopathies, including Thr181, Ser202, Ser205, Thr231, Ser396 and Ser404 [106, 110, 111]. The level of phosphorylated tau decreases into adulthood, where the average level of phosphorylation per tau protein is approximately 2 mol [112] compared to 7 mol in the fetal brain [110]. Tau phosphorylation is also increased in hibernation [113] and hypothermia [114], which is likely due to changes in kinase and phosphatase activity as a result of metabolic alterations. The presence of phosphorylated tau in physiological and developmental conditions reinforces the notion that tau phosphorylation is not merely a result of disease or trauma, but rather that it plays an important role in regulating tau function and localization. It is the aberrant phosphorylation of tau and the inability of cellular mechanisms to maintain these levels in a regulated fashion that can initiate diseased states.

Under pathological conditions, the phosphorylation of tau at certain sites, most notably in the MTBD and C-terminal domain, results in its dissociation from microtubules [115]. The addition of negatively charged phosphates disrupts the ability of tau to bind to microtubules, which in turn can contribute to pathogenesis at two levels. First, it reduces microtubule stability, leading to the disassembly of microtubules and ultimately affecting axonal integrity and axonal transport [116]. Secondly, unbound phosphorylated tau has an increased propensity to aggregate [65, 112, 117]. When tau is abnormally phosphorylated, its ‘paperclip’ conformation is altered, which can further contribute to tau phosphorylation and aggregation [118]. Specific consensus phosphorylation sites have been implicated in having a significant effect on tau aggregation, including Thr175, Thr181, Ser202, Ser205, Thr231, and Ser404 [119–123]. On average, pathological tau contains 6–8 mol of phosphate per protein, nearly three times the normal level [112]. Upon phosphorylation, tau aggregates into soluble oligomers, which are in fact believed to be the most toxic tau species with the ability to spread and seed other aggregates [124, 125]. Ultimately this leads to the formation and aggregation of insoluble filamentous structures, most notably paired helical filaments (PHF) or straight filaments, which are the core components of NFTs that are seen in advanced tauopathies [76].

#### Tau kinases

Phosphorylation is a regulated mechanism governed by the action of kinases which add phosphates to specific amino acids, including serine, threonine and tyrosine. The activation and up-regulation of major kinases are controlled by complex cellular signalling pathways, which typically involve the phosphorylation of their resident catalytic domain. These cellular signalling pathways are often up-regulated in neurodegenerative diseases leading to the activation of kinases and subsequent tau phosphorylation. Three major classes of kinases have been shown to phosphorylate tau. The first class are proline-directed kinases which notably include GSK3β, cyclin-dependent kinase 5 (CDK5), and the mitogen-activated protein kinase (MAPK) family (including extracellular signal-regulated kinase (ERK), c-Jun N-terminal kinase (JNK) and p38). Proline-directed kinases are the most common tau kinases, recognizing and phosphorylating serine and threonine residues that precede a proline. The second class is the non-proline directed kinases, including tau-tubulin kinase, microtubule affinity-regulating kinase, and cAMP-dependent protein kinase (PKA). These kinases phosphorylate serine or threonine residues that do not precede a proline. Lastly, tyrosine kinases, including Src kinases such as Fyn, have been shown to phosphorylate tau at tyrosine residues. For the sake of this review, we will briefly discuss only the proline-directed kinases as they are the most common and widely studied tau kinases. For a comprehensive review of tau kinases, see Martin et al. (2013) [126].

#### GSK3β

GSK3β is a ubiquitously expressed kinase that is involved in a diverse set of cellular functions including cellular metabolism, immune signalling, and neural development [127]. GSK3β was one of the first kinases shown to phosphorylate tau and is classically considered the primary tau kinase [128]. In vitro studies have demonstrated the ability of GSK3β to phosphorylate over 40 residues of tau [121, 129–132], most of which are present in tauopathies such as Alzheimer’s disease and CTE, including Thr231, Ser202 and Ser205. GSK3β expression is increased in human Alzheimer’s disease and colocalizes with NFTs [133, 134]. The expression of GSK3β is also increased in animal models of TBI [135], suggesting that it may play a critical role in the pathophysiology of CTE. Overexpression of GSK3β in cell culture [136, 137] and animal models [138, 139] results in increased tau phosphorylation and pathological fibril formation in addition to other pathologically relevant markers including neuroinflammation and apoptosis. Due to its consensus role in tau phosphorylation and dysfunction, GSK3β has been extensively targeted for therapeutic approaches in neurodegenerative diseases. For example, lithium treatment in animal models of tauopathy or TBI has shown some efficacy in reducing tau phosphorylation and pathology by inhibiting GSK3β [138, 140–142].

#### ERK

ERK was the first identified member of the MAPK family [143] and is the most widely studied. There are two isoforms, ERK1 and ERK2, both of which are expressed in the brain and share an 83% homology [144]. While ERK has been shown to phosphorylate tau, it is primarily involved in cell growth, proliferation and survival [145]. ERK is induced by the traditional MAPK signalling cascade in which extracellular signals activate a series of upstream MAPK kinases (MKKs). ERK is typically activated by mitogenic stimuli such as growth factors and in response phosphorylates downstream targets involved in cell proliferation and survival [145, 146]. However, under certain conditions such as cellular stress and disease, ERK signalling can become dysregulated [147]. Notably, oxidative stress, which has been shown to increase tau phosphorylation, is associated with ERK activation [148–150]. While ERK signalling is classically viewed as pro-survival, chronic signalling has been shown to contribute to neurodegenerative disease as a result of impaired cell survival signalling, but also possibly in part due to its ability to phosphorylate tau. ERK1/2 can phosphorylate tau at over 15 sites in vitro [151–155]. Its activity and expression are increased following TBI [156] and in tauopathies, including CTE [157–159].

#### JNK

JNK is a second subclass of the MAPK family and exists in three isoforms, with JNK1 and JNK2 ubiquitously expressed, whereas JNK3 is preferentially expressed in the brain [160]. JNK is activated upon cellular stress and is primarily involved in mediating apoptotic cell death mechanisms [161]. Thus, dysregulation of JNK signalling has been implicated in the pathophysiology of neurodegenerative disease. In CTE and related tauopathies, JNK activity is increased and colocalizes with pathological tau [157, 159]. JNK can phosphorylate approximately 10 unique residues of tau, most of which are present in neurodegenerative disease-associated tau inclusions [155, 162–164]. In rodents and primary cells, administration of D-JNK1-1, a cell-permeable JNK1 inhibiting peptide, resulted in decreased AT8 (pSer202/pSer205) immunoreactivity [165], further supporting the role of JNK in tauopathy pathogenesis. More recent studies have also suggested that the presence of pathological Aβ may drive JNK activity, which in turn can lead to increased tau phosphorylation [166].

#### p38

As with JNK, p38 is also a subclass in the MAPK family that is activated upon cellular stress and in response to pro-inflammatory stimuli [167–169]. p38 can target various cytoplasmic and nuclear substrates, inducing a diverse set of cellular functions and signalling pathways [167]. There are four p38 kinases with high homology, p38α, p38β, p38γ and p38δ, which are canonically activated by MKK3, MKK4 and MKK6 [170]. p38α and p38β are the predominant isoforms expressed in the CNS, while p38γ and p38δ have tissue specific expression [171]. With respect to neurodegenerative disease pathogenesis, p38 responds to oxidative stress and inflammatory signals which are associated with increased tau phosphorylation. Both in vitro and in vivo studies have demonstrated that increased p38 activity results in increased tau phosphorylation [155, 163, 172], while inhibition of p38 can reduce tau-associated pathogenesis [173, 174].

#### CDK5

CDK5 is highly expressed in the CNS, primarily in neurons, where it plays a critical role in neuronal functioning and development, including neurogenesis, synaptic plasticity, and homeostasis [175]. However, CDK5 is also a crucial mediator in the development of neurodegenerative disease, whereby aberrant activation can lead to pathological tau phosphorylation, Aβ production, mitochondrial dysfunction and cell death [176, 177]. The activation of CDK5 through the binding to p25 is upregulated in Alzheimer’s disease [178] and following TBI [179, 180]. Overexpression of p25, leading to increased CDK5 activity, resulted in tau hyperphosphorylation and NFT development [178, 181, 182]. Further, CDK5 can phosphorylate and subsequently activate other tau kinases, such as GSK3β and ERK, which may further exacerbate tau pathology [183].

#### Phosphatases

In addition to the phosphorylation of tau by kinases, the dephosphorylation of tau by protein phosphatases is also a critical component of the pathophysiology of tauopathies [184]. An imbalance in the expression and activity of protein kinases and phosphatases has been implicated as an important factor in the development of tauopathies [185]. The main phosphatase involved in the dephosphorylation of tau is protein phosphatase-2 A (PP2 A), which is a serine/threonine phosphatase. PP2 A accounts for approximately 71% of tau phosphatase activity [186]. Under physiological conditions, PP2 A localizes to axons and dendrites and, in concert with tau kinases, controls the level of tau phosphorylation in a delicate balance. It has been suggested that proper PP2 A activity and its binding to tau are essential for appropriate tau localization. However, in neurodegenerative states PP2 A is downregulated, in some instances by nearly 50%, causing a shift in the physiological regulation of phosphorylation and ultimately leading to increased tau phosphorylation [186, 187]. In CTE, genes encoding protein phosphatase subunits were significantly downregulated and were associated with increased p-tau [157]. Similarily, PP2 A was decreased in experimental models of TBI [188, 189]. Inhibition of PP2 A by toxins, including okadaic acid [190, 191] or by endogenous inhibitors such as inhibitor-1 of PP2 A (I_1_^PP2 A^) [192, 193]_,_ results in increased tau phosphorylation. PP2 A may also indirectly influence tau phosphorylation by modulating the activity of tau kinases GSK3β, ERK, and JNK [190, 194, 195]. Targeting PP2 A as a therapeutic approach in tauopathies has been examined but due to its diverse set of functions beyond tau phosphorylation, it presents particular challenges and considerations.

#### Tau phosphorylation in CTE

**Fig. 3:**
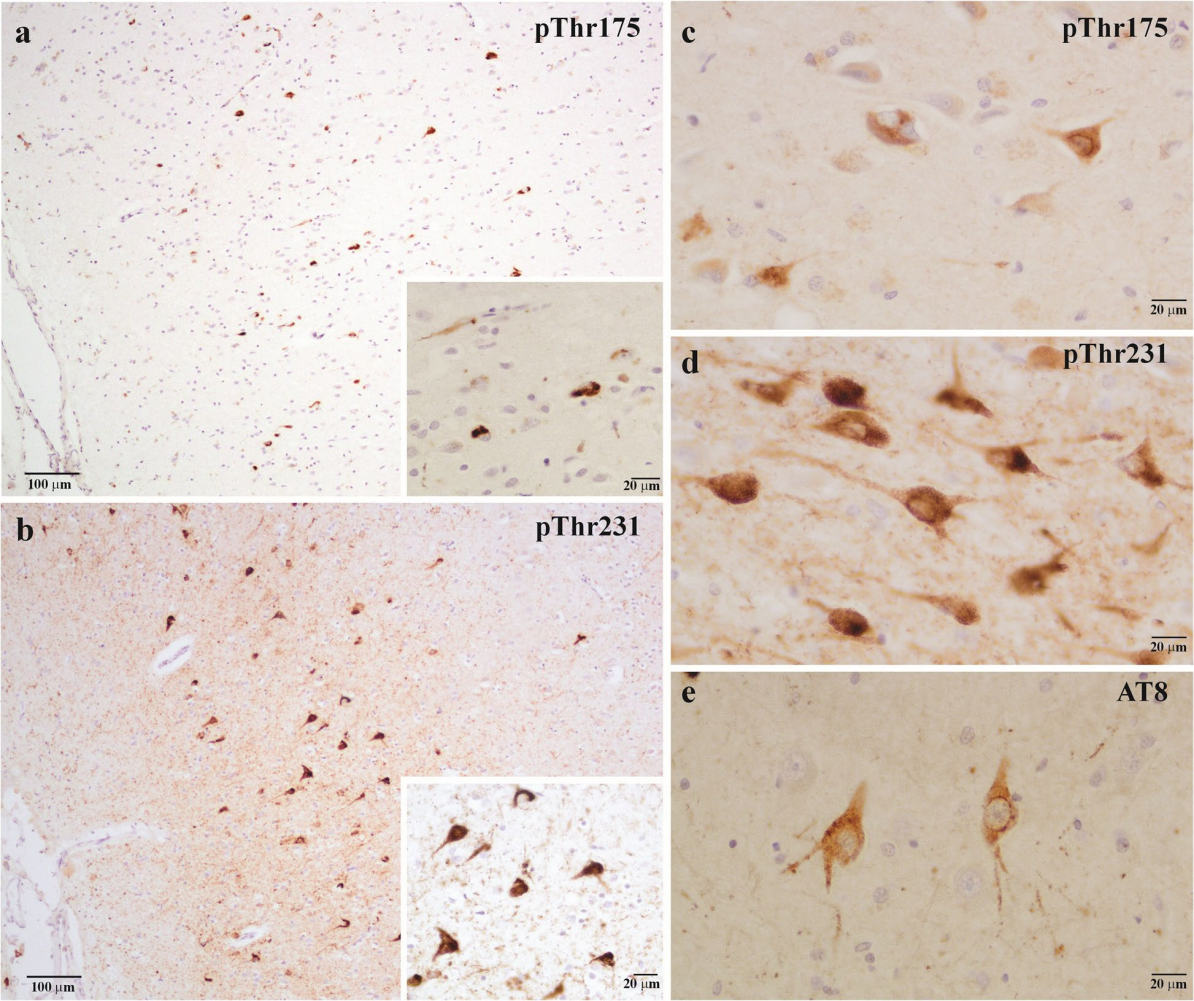
CTE tau pathology. **a** Neuronal pThr175 tau staining in the temporal cortex at depth of the sulcus. **b** Pathognomonic perivascular pThr231 tau immunoreactive neurons in the temporal cortex. NFT-bearing neurons in the hippocampus stained for (**c**) pThr175, (**d**) pThr231 and (**e**) AT8 tau

In CTE, aggregated p-tau deposits present as the major neuropathological hallmark necessary for post-mortem diagnosis (Fig. 3). In CTE, phosphorylated tau accumulates as intracellular NFTs or glial tangles [196], as seen in other tauopathies. However, CTE is unique from other primary tauopathies in that p-tau inclusions are mainly restricted to the perivascular space around blood vessels and the depths of the cerebral sulci [24, 196]. This is likely due to the nature of these brain structures and regions being the most vulnerable to sheer physical damage [197]. Phosphorylated tau inclusions are also seen in superficial cortical layers II and III, which is not typical of Alzheimer’s disease [19, 196]. This unique distribution of tau is now considered pathognomonic for CTE. Pathological tau inclusions are found primarily in neurons as NFTs or neuropil threads, and, to a lesser extent, in astrocytes as astrocytic tangles [196, 198]. In CTE, neuronal p-tau inclusions typically consist of 3R and 4R tau, while astrocytic tangles are predominantly 4R tau isoforms [199].

Tau pathology in CTE can range in severity from mild with isolated p-tau lesions in the neocortex, to severe, which is characterized by widespread p-tau in the temporal lobe, forebrain, and brainstem [200]. Tau pathology in CTE demonstrates a hierarchical progression throughout the brain, spreading to functionally and anatomically connected regions with an increased p-tau load. In an attempt to more accurately classify the CTE severity, McKee et al. [8] proposed staging criteria that classifies CTE as one of four stages based on p-tau burden and anatomical deposition [8]. Stage I CTE is defined by p-tau as NFTs and astrocytic tangles in the cortical perivascular and the sulci, typically in the dorsolateral frontal cortex. Stage II is defined by the progression of tau pathology into the temporal and parietal cortices, with sparse NFTs in the superficial layers of the cortex. In Stage III CTE, p-tau lesions are distributed throughout the cortex, including the superficial layers II and II, and progress into the hippocampus, entorhinal cortex, amygdala, and substantia nigra. Stage IV CTE is characterized by severe tau pathology in the form of widespread NFTs and ghost tangles throughout the cerebral cortex and temporal lobes, accompanied by extensive neurodegeneration and gliosis. As per the McKee staging criteria, the deposition pattern of tau in CTE differs from the stereotypical Braak staging of tau in Alzheimer's disease, where the earliest pathology is observed in the entorhinal cortex and spreads to the temporal lobes and neocortex [201]. Despite the McKee staging scheme being independently validated [202], the second NINDS/NBIB meeting to define the neuropathological criteria for CTE was unable to reach a consensus agreement on the McKee stages for assigning CTE severity and thus proposed a binary classification system as either low CTE (mild) or high CTE (severe) [19]. A scoring system is utilized in which the presence of p-tau in different anatomical regions, such as the gyral bank and crest, cortical layer II, CA2 and CA4 hippocampal subregions, entorhinal cortex, amygdala, thalamus, mammillary body and cerebellar dentate nucleus, is assigned a value of one. Upon neuropathological examination of paraffin-embedded tissue, a score of less than five is classified as low CTE and a score of five or more is classified as high CTE.

As previously mentioned, tau is phosphorylated at over 50 unique epitopes in tauopathies, with specific phospho-epitopes including Thr175, Thr181, Ser202, Ser205, Thr217, Thr231, Ser396, and Ser404 being critical in driving tau aggregation. These sites primarily reside in the proline-rich or C-terminal projection domain and are present in nearly all NFTs and p-tau immunoreactive inclusions. In late-stage CTE, pSer202, pThr231, and pSer396 are all significantly increased compared to controls and early-stage CTE [203]. Moreover, the ratio of pSer202 to pSer396 is significantly greater in CTE compared to Alzheimer’s disease. Taken together, this suggests that the pathogenesis of phosphorylated tau in CTE may differ from other tauopathies, including Alzheimer’s disease. Interestingly, pThr181 tau is decreased in the frontal cortex of late-stage CTE compared to controls and Alzheimer’s disease [203]. In contrast, extracellular vesicles derived from CTE patients had enriched levels of pThr181 tau compared to age-matched controls [204]. Phosphorylation of other sites are increased in CTE including Thr175 [135], Ser199 [157], Ser422 [205], and oligomeric tau (Tau Oligomeric Complex 1; TOC1) [205].

In some cases, the phosphorylation of tau at specific sites induces conformational changes including tau truncation, N-terminal PAD exposure, and isomerization, which further potentiates tau aggregation. Pseudophosphorylation of Thr175 in vitro induces PAD exposure, which subsequently results in GSK3β activation, Thr231 phosphorylation, and fibril formation [58, 206]. This mechanism was recapitulated in an experimental model of TBI that displays CTE-like pathology [135]. A separate study showed that the phosphorylation of Thr231 tau in CTE and experimental TBI is associated with an increase in a toxic isomer of *cis* p-tau [207]. The proline isomerase Pin1 converts *cis* p-tau to *trans* p-tau, which is a non-pathogenic conformer that promotes tau assembly [208]. *Trans* p-tau can be dephosphorylated by PP2 A and is less prone to aggregation [209, 210], but decreased levels of Pin1 activity in neurodegenerative disease and TBI promote an increase in *cis* p-tau [208, 211, 212]. This increase in pathological *cis* p-tau that occurs following TBI can be mitigated by the administration of a monoclonal antibody targeting *cis* p-tau, suggesting a potential avenue for reducing tau pathology [207].

#### Experimental models of TBI and tau

To examine the complex nature of both TBI and tau pathophysiology, experimental animal models have been developed to help better understand the underlying molecular mechanisms. Experimental TBI has been applied to many different species including rodents, pigs, cats, ferrets and monkeys. As with any experimental model, there are important limitations to consider when making evidence-based conclusions and applying them to humans.

Various models of experimental TBI exist, including controlled cortical impact (CCI), fluid percussion impact (FPI), weight drop model and blast injury model (reviewed in [213]). These models typically involve placing the head in a fixed position, exposing the dura by craniotomy and delivering a direct impact to the brain [214]. Traditional experimental TBI models result in mild to severe TBI, with apparent cortical damage and evidence of a pronounced cellular response, including neuroinflammation, neurodegeneration and tau pathology (Table 1). These models allow for precise control over parameters such as speed, depth, and dwell time. However, due to the severity and invasiveness of the injury, these models often only involve a single hit and thus do not properly capture the repetitive nature of impacts that are the primary risk factor for CTE, nor do they model the true subconcussive impacts suffered by most individuals who develop CTE [215]. To address these limitations, closed head injury (CHI) models have been developed which better replicate the physical characteristics of a typical mTBI. Some studies have modified earlier models, such as the weight drop or CCI model, by eliminating the craniotomy and performing the impact on an intact skull. The CHIMERA model is one which has modified the CHI model to allow for the ability of the head to move freely upon impact and produce acceleration and rotational forces, a key aspect of TBI [216, 217]. Moreover, CHI models allow for repetitive impacts, which more closely resembles the nature in which mTBIs are sustained in people who develop CTE. Regardless of the recent improvements and considerations surrounding the models of TBI, there remain critical limitations from a neuropathological standpoint. While tau pathology has been described in TBI animal models, the stereotypical neuronal and glial p-tau pathology adjacent to the vasculature that is characteristic of CTE is not observed in rodents. Additionally, tau pathology at the depths of sulci is not reproducible due to their lissencephalic brain.

**Table 1:** Rodent models of TBI

Model	Parameters	Animal model	Tau pathology	Additional pathology	Reference
**Controlled cortical impact (CCI)**	5.0 m/s speed 100 ms dwell 1.5 mm depth	hTau mice (5–7 months)	pSer199, pSer202/Thr205 (AT8), pSer214, pSer262/Ser356, pSer396/Ser404 (PHF-1)	Synapsin, Synaptophysin, PSD-95, NeuN, GFAP	[218]
	3.5 m/s speed 500 ms dwell 2.0 mm depth	Sprague Dawley rats (3–4 months)	pThr175, pThr231, PAD (TNT1)	pTyr216-GSK3β, GFAP, Iba1	[135, 219]
	5.0 m/s speed 100 ms dwell 2.0 mm depth	3xTg-AD mice (6 months) APP/PS1 mice (2 months) P301L-Tau mice (6 months)	Total tau, pSer199, pSer396/Ser404 (PHF-1)	Aβ	[220]
	3.5 m/s speed 100 ms dwell 2.0 mm depth	C57BL/6 mice (2–3 months)	pThr205, pSer262, pSer404	APP, NeuN	[221]
	1.0 mm depth (mild) 1.5 mm depth (mild-moderate) 2.0 mm (moderate)	3xTg-AD mice (5–7 months)	pSer199, pSer202/Thr205 (AT8), pSer212/Thr214 (AT100), pThr231, pSer396, pSer422	Aβ, Aβ_42_, Aβ_40_, APP	[222]
	3.5 m/s speed 1.0 mm depth (mild) 1.5 mm depth (mild-moderate) 2.0 mm (moderate)	Sprague Dawley rats	Cleaved tau-7		[223]
**Fluid percussion injury (FPI)**	4.8 kg steel pendulum	Sprague Dawley rats	pSer202/Thr205 (AT8), pThr231 (AT180), Oligomeric tau (T22)		[224]
	3.0 atm pressure	Long-Evans rats (3 months)	pSer198, pSer262	PP2 A	[225]
	1.0 atm pressure	hTau mice (2 months)	pThr231 (AT180)	CD45, CD68	[226]
**Weight drop**	400 g weight 2.5 cm height	C57BL/6 mice (2–3 months)	pThr231	NeuN	[227]
	82 kPa (peak overpressure) 71 kPa (maximal impulse)	C57BL/6 mice (2 months)	pThr181 (AT270)	Silver stain	[228]
	17–22 psi	Sprague Dawley rats	Oligomeric tau (T22)		[229]
**Blast**	108.9 kPa 15.8 psi	C57BL/6 mice (3–4 months)	pSer202, pSer396, pThr181, pSer212/Thr214 (AT100), Cleaved tau (TauC3)	SOD2, GFAP	[230]
	77 ± 2 kPa 4.8 ms duration 1.26 Ma	C57BL/6 mice (2.5 months)	pThr181 (AT270), pSer202 (CP-13), pSer199, pThr205	GFAP, SMI-31	[27]
**Repetitive TBI**	5x 3.5 m/s speed 500 ms dwell 1.0 mm depth	C57BL/6 mice (2–2.5 months)	pSer202/Thr205 (AT8)	APP, GFAP, Iba1, TNF-α, IL-6, PSD-95, Synaptophysin	[231]
	2x/week (3 or 4 weeks) 5.0 m/s speed 200 ms dwell 1.0 mm depth	hTau mice (4 months)	pThr231, Oligomeric tau (TOC 1)	APP, GFAP, Iba1	[232]
	1x, 3x, 5x 54 g weight 96 cm height	3xTg-AD mice (2–4 months)	pSer262/Thr263, pThr181	GFAP, S100β, CD68, TMEM119, Aβ_40_, Aβ_42_, NeuN, IL-1, IL-9, IL-17, pAft2, pMek1/2	[233]
	7x 54 g weight 28 in height	C57BL/6 mice (2–3 months)	*cis* pThr231 tau		[207]
	5x 50 g weight 15 cm height	C57BL/6 mice (2–3 months)	pSer202/Thr205 (AT8), pThr231 (AT180)	GFAP, Iba1, TDP-43, MBP	[234]

In addition to the limitations of animal models of TBI, investigating tau biology in rodents introduces additional caveats. In contrast to humans who express the full complement of all six tau isoforms throughout adulthood, rodents predominantly express 4R tau isoforms, whereas 3R isoforms are highest during early development and decrease with aging [235, 236]. While the exact effect of differences in tau isoform expression is not completely understood, CTE is a known mixed tauopathy with both 3R and 4R isoforms pathologically altered [46]. Moreover, 3R isoforms play an important role in tau physiology, such as in the nucleus, which although not fully understood in the context of TBI or disease, may contribute to tau pathophysiology [89]. To address this confound, transgenic rodent models expressing humanized tau isoforms have been developed and recently utilized in experimental TBI [226, 232]. The first-generation models expressed various isoforms of human tau under different promoters [237, 238]. More recently, *MAPT* knock-in models have been generated, which replace the entire murine *MAPT* gene and express all six human tau isoforms [239]. Despite these advances, animal models expressing either murine or human tau still do not naturally develop tau pathology with aging, as seen in higher-order mammals and humans.

To better investigate the pathogenicity of tau and tauopathies, animal models overexpressing known tau mutations have been developed, such as the FTD mutations P301L and P301S [240–245]. These models reliably produce robust NFT tau pathology and thus serve as valuable experimental tools to investigate characteristics of tau pathology and pre-clinical interventions [246, 247]. However, these models may not always present with the same regional or cell-type specificity as seen in human tauopathy, often express only one isoform, and can also introduce off-target effects in the coding sequences of other genes [248, 249]. The use of transgenic animal models that express genetic variants limits the relevance of examining sporadic tauopathies. In addition, given what we now know about the heterogeneity of tauopathies with respect to structural conformation and neuroanatomical deposition, the predominant use of transgenic models harbouring FTD-MAPT mutations for investigating different tauopathies may limit the translational potential and is an important consideration when selecting the appropriate model. Despite these limitations, the ability to generate novel transgenic animal models with multiple genetic modifications allows for the investigation into the role of other factors that may contribute to tau pathogenesis in CTE, such as APOE status or TMEM106B variants.

#### Pathological tau strains

The aggregation of tau from a monomer to an insoluble NFT is common amongst all tauopathies. While it was classically believed that all NFTs adopted a similar conformation or strain, this viewpoint has been recently challenged, stating that individual tauopathies differ in the conformational folding of tau fibrils. Advancements in cryo-EM have demonstrated that CTE tau folds in a unique structural conformation compared to other tauopathies such as Alzheimer’s disease, Pick’s disease, PSP or in vitro tau aggregates [250, 251]. In CTE, tangles are composed primarily of CTE Type I and II filaments, which are distinct from PHFs and straight filaments observed in Alzheimer’s disease and other tauopathies [250]. CTE-tau filaments contain a unique hydrophobic cavity that is formed by the β-helix region, which encloses an unknown density that is not seen in Alzheimer’s disease tau filaments [250]. This may suggest that the incorporation of additional factors is important in the aggregation of tau fibrils in CTE. It had been speculated that the inclusion may be a cofactor, such as non-polar sterols or fatty acids, that promotes a unique folding confirmation of tau. Recently, in vitro assembly of CTE-like tau filaments required the presence of NaCl, suggesting the unknown density may be inorganic salts [252]. Regardless, it is conceivable that this density may be related to the nature of TBI and the unique underlying cellular responses that are associated with it. Overall, this finding reinforces that not all tauopathies are identical, and the differences in the pathological tau strains may explain the clinical diversity amongst tauopathies.

It has also been demonstrated that the aggregation and propagation of tau differs among tauopathies. The transmission of tau strains from Alzheimer’s disease, PSP or CBD into non-transgenic mice resulted in strain-specific differences in the ability to seed tau aggregation and cell-type specificity [253]. In line with this, tau isolated from either Pick’s disease (a 3R tauopathy), PSP and CBD (a 4R tauopathy) or CTE and Alzheimer’s disease (a 3R/4R tauopathy) was only able to induce tau aggregation in cells stably expressing their respective tau isoform [254]. These findings suggest that the difference in disease pathogenesis and phenotype may be related to the underlying mechanisms that initiate tau pathogenesis uniquely in each disease context. Understanding the differences between tauopathies and why tau strains differ is critical for developing an accurate diagnostic biomarker and treatment specifically for CTE. Of interest, it has been shown that tau filaments from Guamanian variants of amyotrophic lateral sclerosis/parkinsonism-dementia complex (ALS-PDC) adopt a similar conformational folding to that of CTE p-tau, most notably by the presence of the unknown nonproteinacious inclusion within the β-helix cavity [255]. ALS-PDC is a rare disease characterized histopathologically by tau, Aβ, alpha-synuclein, and TDP-43 inclusions [256]. While its etiology remains unknown, it is speculated to be caused by environmental factors. The commonality of environmental and exogenous factors playing a role in both CTE and ALS-PDC may underlie the similarity of tau strain between the two diseases in comparison to other tauopathies. Moreover, ALS is one of the most common comorbidities in CTE patients, and over 80% of CTE patients also present with concomitant TDP-43 pathology in late stages [9, 257]. Further, we have previously shown that phosphorylated Thr175 tau is present in CTE and CTE-ALS patients, suggesting a common pathophysiological link between the diseases [135].

#### Mechanisms contributing to tau phosphorylation in TBI and CTE

**Fig. 4:**
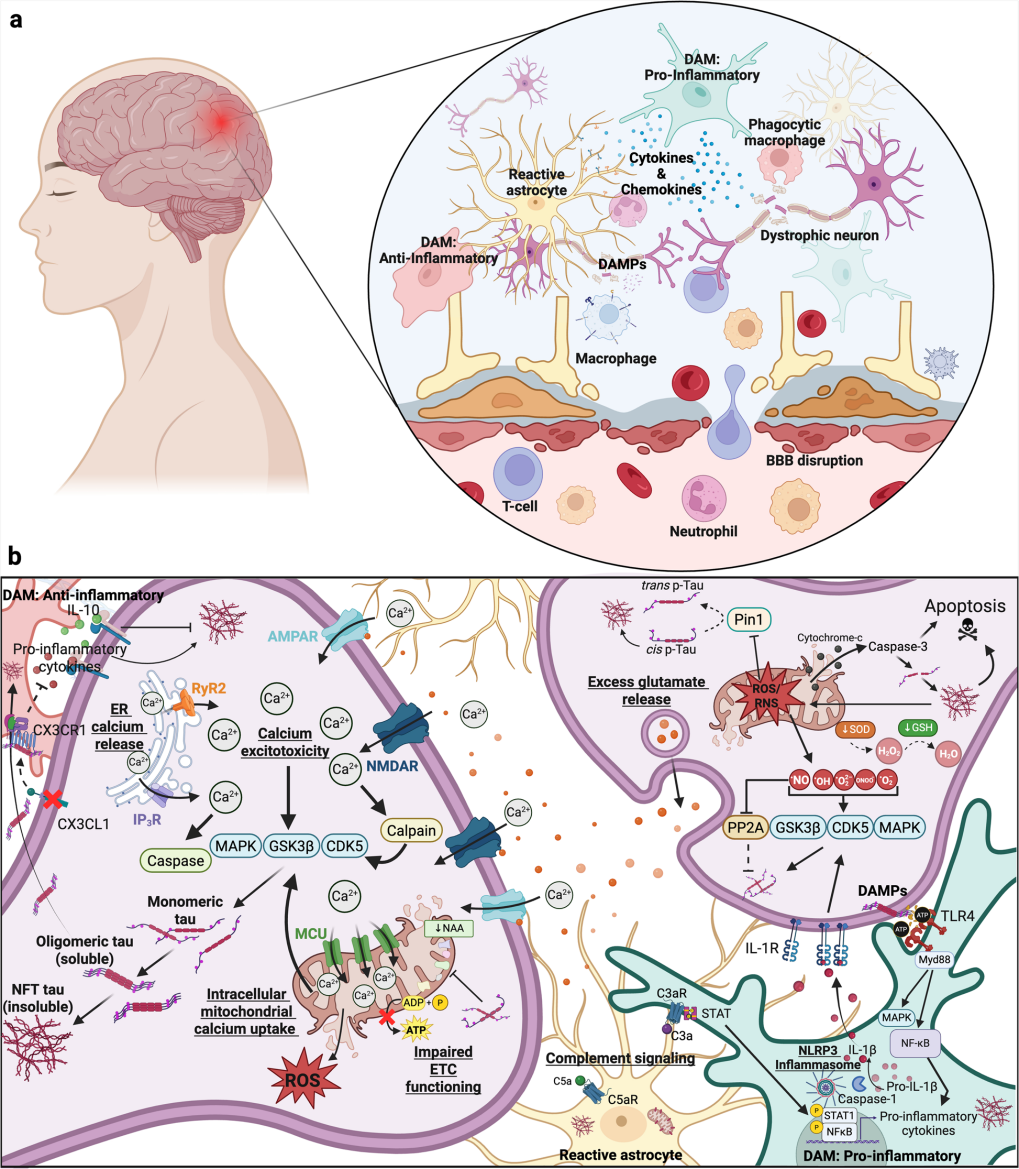
Proposed cellular mechanisms of tau phosphorylation in TBI and CTE. **a** Traumatic brain injury (TBI) is characterized by an initial primary injury response, in which increased mechanical forces and strain lead to axonal shearing and blood–brain barrier disruption. In response, innate and peripheral immune cells migrate to the damaged region where they sense disease-associated molecular patterns (DAMPs) and facilitate tissue healing mechanisms. **b** Microglia and astrocytes respond to DAMPs released from dystrophic neurons and become activated accordingly. Microglia take on a pro- or anti-inflammatory molecular signature characterized by the release of various cytokines and the expression of certain receptors. Activation of the complement cascade and NLRP inflammasome stimulate cytokine and chemokine production through intracellular STAT and NFκβ signaling pathways. Cytokine signaling can lead to the activation or inhibition of kinases, which can subsequently phosphorylate tau and lead to soluble oligomeric and insoluble neurofibrillary tangle (NFT) aggregation. Excitotoxicity is characterized by an increase in intracellular calcium through NMDA and AMPA receptor-mediated membrane permeability driven by excess synaptic glutamate release, and ryanodine receptor (RyR) and inositol 1,4,5-trisphosphate receptor (IP_3_R)-mediated ER calcium efflux. Calcium excitotoxicity can lead to kinase, caspase and calpain activation, which increases p-tau. In response, the mitochondria rapidly uptake cytosolic calcium leading to mitochondrial dysfunction that is characterized by a reduction in ATP synthesis, increased production of reactive oxygen species (ROS), and the release of apoptotic factors, such as cytochrome-c. Increased ROS production and decreased antioxidant function lead to oxidative stress, which activates kinases, inhibits phosphatases and modulates Pin1 activity. CX3 CL1 signaling is downregulated, resulting in increased pro-inflammatory signaling and internalization of soluble tau through the CX3 CR1. MAPK: mitogen-activated protein kinase. GSK3β: glycogen synthase kinase 3 beta. CDK5: cyclin-dependent kinase 5. NMDA: N-methyl D-aspartate. AMPA: 2-amino-3–5-methyl-4-isoxazoleproprionic acid. STAT: signal transducer and activator of transcription. NF-κB: Nuclear factor-kappa B. IL: Interleukin. TLR: Toll-like receptor. NAA: N-acetyl aspartate. Created in BioRender

The mechanisms leading to the unique and stereotypical deposition and distribution of tau pathology in CTE compared to other tauopathies are not fully understood but have been attributed to the complex cellular mechanisms that result from exposure to repeated head impacts and TBI (Fig. 4). The pathophysiology of TBI includes an initial primary injury response which is associated with physical and mechanical damage due to forces produced by the TBI itself [3]. This is followed by a secondary injury response that is characterized by cellular and molecular alterations which can last for extended periods of time [3]. It is believed that the chronic and long-term cellular changes attributed to the secondary injury response are primarily responsible for the aberrant phosphorylation of tau and the development of CTE. This is supported by the current consensus that repetitive head injuries, irrespective of a formal concussion, are required for the development of CTE, while a single TBI has rarely been demonstrated to cause CTE [96, 258]. Repeated subconcussive injuries are highly associated with increased risk for CTE and have been shown to induce complex cellular and molecular changes including neuroinflammation, excitotoxicity, impaired mitochondrial functioning, production of reactive oxygen species, and induction of oxidative stress [3]. It is important to note that these mechanisms are often related and can act synergistically, making them challenging to study in isolation. Understanding the role of these cellular processes is increasingly critical in developing diagnostic biomarkers and therapeutic targets, especially considering attempts to target p-tau itself have failed in clinics.

#### Primary mechanical injury

TBI can range from mild to severe and can be characterized as focal or diffuse. In general, moderate and severe TBI induce physical damage to the underlying brain tissue. Moreover, damage from focal TBI is typically restricted to the immediate brain regions but is more pronounced and is often associated with intracranial hematoma and hemorrhage [259]. There are four types of intracranial hemorrhages which include epidural, subdural, subarachnoid, and intracerebral. In some cases, if severe enough or left untreated, intracranial hemorrhaging can result in death [260, 261]. Diffuse TBI presents with more widespread axonal injury, which includes the shearing of axons and in some cases, damage to the surrounding blood vessels and blood–brain barrier [262, 263]. This is typically a result of the translational or rotational forces produced and the resultant mechanical strain generated.

Computational modelling of TBI demonstrated that mechanical strain and strain rate are most prominent at the depths of the sulcus, and thus provides reasoning as to why p-tau in CTE is primarily restricted to the sulci [264]. This immediate mechanical and physical injury has been demonstrated to result in necrotic cell death [265, 266]. While mechanical injury caused by head trauma is unlikely to directly cause significant tau phosphorylation, it is critical in leading to the secondary injury response, and in turn leading to the pathogenesis of p-tau. One study found that axonal injury and white matter degeneration, as measured with diffusion tensor imaging in CTE post-mortem tissue, are significantly correlated with the level of p-tau [267]. Controlling the effects of primary mechanical injury acutely following TBI is critical in reducing the long-term cellular effects [268, 269] and may lower the risk of developing CTE. Identifying these changes through the use of imaging modalities, such as functional magnetic resonance imaging, can help diagnose the severity of brain trauma and provide insight into the proper medical care required to reduce the effects of the secondary injury.

#### Neuroinflammation

Following TBI, a neuroinflammatory response is rapidly initiated in which immune cells migrate to the region of interest where they release various inflammatory mediators in an attempt to minimize the damage and begin repairing tissue. In the short term, neuroinflammation is carried out as a protective mechanism, repairing synapses, recycling damaged cells, and phagocytosing dystrophic neurites and tau burdened synapses [270–273]. However, prolonged activation of the immune system can result in chronic neuroinflammation and lead to the induction of neurodegenerative signaling pathways [274, 275]. Chronic neuroinflammation is a hallmark of CTE, caused by repetitive exposure to TBI, chronically upregulating immune signaling and in turn increasing tau pathology [276]. Fluid biomarker studies have demonstrated that various markers of immune activation are upregulated in TBI and CTE patients, including cytokines interleukin (IL)−1β, IL-6, IL-10; chemokines CXCL6, CXCL10, CCL11 [277–280]; and cell surface receptors such as triggering receptor expressed on myeloid cells 2 (TREM2) [281] and CX3 CR1 (fractalkine receptor) [282]. Increased levels of immune markers post-TBI are most often associated with worsened prognostic outcomes and, in some cases, more severe p-tau pathology and neurodegeneration [276].

Microglia and astrocytes are the primary cells of the innate immune system, where they act as one of the key first responders and main drivers of the initial neuroinflammatory response. Microglia and astrocytes have both been widely implicated in mediating neuroinflammation, tau dysfunction, and neurodegeneration in tauopathies [276, 283]. Aberrant glial activation can induce tau phosphorylation in neurons through cytokine signaling and kinase activation [284]. Microglia and astrocytes also play a key role in the seeding and spreading of tau aggregates throughout the CNS [285–289]. It has been demonstrated that activated glia can engulf and phagocytose neuronal synapses that harbour tau aggregates [273]. Less is understood about the role of astrocytes in the context of tauopathies. However, evidence of tau-bearing astrocytes is a key pathological feature of CTE [196] and levels of GFAP are increased in the cerebrospinal spinal fluid (CSF) of patients with a history of concussions [290].

Upon exposure to TBI, microglia and astrocytes are recruited to the site of injury through the recognition of disease-associated molecular patterns (DAMPs), which are released from damaged or dying cells [291, 292]. Damaged neurons release DAMPs, such as lipids, mitochondrial DNA, ATP, and proteins, including high mobility group box protein 1 (HMGB1) and S100B, which bind to pattern recognition receptors, including toll-like receptors (TLRs) and NOD-like receptors (NLRs) [292–295]. Upon binding, downstream transcription factors are activated and stimulate the production and release of various inflammatory cytokines, chemokines, and reactive oxygen species (ROS). Through the expression of various cytokines and chemokines, microglia transition from a resting-homeostatic state to an active or reactive state [296, 297]. Despite traditionally classifying microglia as either pro-inflammatory (M1 phenotype) or anti-inflammatory (M2 phenotype) based on the expression of specific cytokines, chemokines, and receptors, this notion has been challenged in recent years [298]. It is now understood that microglia exist in multiple states as different subpopulations that express unique patterns of receptors and cytokines. Thus, it is more accurate to place microglia reactivity states on a spectrum ranging from pro-inflammatory to anti-inflammatory. Recent advances in single-cell sequencing have demonstrated the complexity of the innate immune response in TBI [299], further underscoring the importance of reclassifying microglia more accurately and appropriately based on specific expressions of immune markers. In fact, the term disease-associated microglia (DAM) has been proposed to encompass a specific subset of activated microglia in diseased states [298, 300, 301]. Using this simplified classification, it has been demonstrated that following TBI, microglia can transition through multiple states over the course of hours to days to weeks, with a prominent pro-inflammatory response upregulated early on followed by a heightened anti-inflammatory response in the more chronic state [302–305].

In TBI, DAMPs bind to TLRs on microglia and activate intracellular effectors including myeloid differentiation primary response 88 (Myd88), signal transducer and activator of transcription (STAT), and nuclear factor-kappa B (NF-κB), which translocate to the nucleus for the transcription of various inflammatory cytokines, including IL-1β, IL-6, IL-10, IL-18 and tumor necrosis factor-alpha (TNF-α) [306]. Tau protein, acting as a DAMP, has also been shown to induce NF-κB activation, in part by directly binding to TLRs, and promote microglial-mediated tau spreading and associated cognitive dysfunction [285, 307, 308]. Inactivation of NF-κB signaling reduced tau-dependent cognitive deficits, partially restored microglia from a DAM state [285], and reduced p-tau, caspase-3 activation, and cleaved-tau [309]. Increased production of pro-inflammatory cytokines has been directly linked to increased p-tau and neurodegeneration [310]. Notable pro-inflammatory cytokines include IL-6, IL-18, TNF-α, and IL-1β. IL-1β is a key pro-inflammatory cytokine that has been demonstrated to increase tau phosphorylation through the activation of kinases, including MAPK and CDK5 [311–314]. Inhibition of IL-1β signaling in animal models of tauopathy reduced p-tau, ameliorated cognitive dysfunction and downregulated MAPK activation [315].

The production and release of IL-1β is driven by the inflammasome, a multimeric protein complex consisting of an NLRP scaffolding protein (NLRP1 in neurons and NLRP3 in microglia), the adaptor protein ASC and the effector protein, caspase-1. The binding of DAMPs and misfolded proteins, including tau aggregates, to TLRs can prime the NLRP3 inflammasome for activation where caspase-1 cleaves Pro-IL-1β into its active form IL-1β, which is then ready to be released [316–318]. The expression of the inflammasome and its protein subunits are increased acutely following animal models of fluid percussion and blast injury [319–322], and in the CSF of patients with TBI [323]. Knockdown and inhibition of the NLRP3 inflammasome in tau transgenic mice reduced hippocampal p-tau and seeding, which was associated with decreased IL-1β levels, and reduced GSK3β and calcium-calmodulin-dependent protein kinase II (CAMKII) activity [316, 317]. Activation of the NLRP3 inflammasome by p-tau aggregates, leading to cytokine release and kinase activation, results in a feed-forward loop in which tau phosphorylation is increased and can further potentiate the neuroinflammatory response.

On the other hand, anti-inflammatory cytokines, notably IL-10 and transforming growth factor beta (TGFβ), work in concert with pro-inflammatory cytokines to regulate the inflammatory response. Anti-inflammatory cytokines promote tissue healing and remodeling, and dampen chronic neuroinflammation by inhibiting pro-inflammatory cytokine production [324–329]. Anti-inflammatory cytokine signaling has also been implicated in tau pathogenesis and TBI. IL-10 knockout in mice administered LPS resulted in increased tau phosphorylation which was directly associated with increased pro-inflammatory cytokine production and p38-dependent tau phosphorylation [330]. Interestingly, two independent studies demonstrated differing results when targeting the TGFβ pathway in animal models of TBI, further underscoring the complexity of cytokine signaling and the opposing roles they may play in physiological and pathological states. Knockdown of TGFβ1 in TBI rats increased neuronal loss and astrogliosis, indicative of increased apoptosis, and worsened cognitive outcomes, suggesting TGFβ1 is neuroprotective following TBI [331]. However, a second study showed that inhibition of the TGFβ receptor reduced neuroinflammation and apoptosis [332]. Chemokines also play a key role in mediating microglial states. CX3 CL1 (fractalkine) is a chemokine produced by neurons, which binds to its complementary receptor CX3 CR1 on microglia and reduces pro-inflammatory cytokine synthesis, dampening glial reactivity [333–335]. Interestingly, it has been demonstrated that soluble tau is able to compete with CX3 CL1 and bind to CX3 CR1 to promote its internalization [336]. Increased levels of CX3 CL1 in the CSF of TBI patients were observed in a limited group, while levels of CX3 CR1 were increased in animal models of TBI [282]. Genetic knockdown of CX3 CR1 in mice expressing human tau accelerated tau pathology that was driven by IL-1R and TLR-induced activation of p38 [337, 338], while upregulating CX3 CL1 expression ameliorated p-tau pathology and related cognitive deficits [339–341]. In the case of TBI, mice lacking CX3 CR1 had less neurodegeneration in the acute phase, but worse outcomes in the chronic neuroinflammatory phase [342].

Another key contributor to the innate neuroinflammatory response post-TBI is the complement signaling cascade, which helps mediate the immune response by opsonizing damaged cells and inducing phagocytosis [343]. Complement signaling results in the formation of the membrane attack complex (MAC) and the recruitment of immune cells through chemotaxis. Components of the complement cascade, including C3, C5, and the MAC, are elevated in TBI patients [344–347] and can induce tau phosphorylation through activation of MAPKs and GSK3β [348, 349]. Dysregulation of the complement cascade has been described in tauopathies and components of the pathway are localized to synaptic densities that contain p-tau in PS19 mice [350]. Targeting the complement cascade has proved to be neuroprotective in animal models of tauopathy and TBI [343]. Genetic deletion of C3 in tauopathy mice mitigated neurodegeneration and neuroinflammation through the reduction of STAT-dependent cytokine production, including IL-1β [351, 352]. Accordingly, IL-1β leads to tau phosphorylation through kinase activation and thus, inhibition of STAT signaling decreased levels of p-tau.

The peripheral immune system also plays a prominent role in providing support to the brain post-TBI and has recently garnered attention for its role in tau pathogenesis and neurodegeneration [353–355]. Following primary injury, secreted chemokines and complement proteins signal to peripheral immune cells such as T-cells, B-cells, neutrophils, monocytes and macrophages to infiltrate the site of injury where they can assist with phagocytosis of debris and upregulation of the neuroinflammatory response [356, 357]. Mice harbouring the P301S tau mutation and human APOE4, which develop severe tau pathology and neurodegeneration, displayed a significant increase in T-cells compared to animals lacking the P301S tau mutation or those with amyloid pathology [353]. Moreover, there was an increase in the percentage of DAM and interferon (IFN)-activated microglia, and targeting both microglia and T-cells prevented tau-mediated neurodegeneration [353]. In line with this, increased neutrophil infiltration was observed in an Alzheimer’s disease animal model that displays tau and amyloid pathology [358]. Given the evidence of the role that the adaptive and innate immune systems play in tauopathy and TBI, targeting mediators of the neuroinflammatory axis may be a potential therapeutic target for reducing p-tau and the progression of CTE.

#### Excitotoxicity and calcium signaling

Excitotoxicity is one of the key secondary injury processes in the TBI sequelae caused by the excessive release of excitatory neurotransmitters, particularly glutamate, resulting in an increase in intracellular calcium accumulation [359–362]. Chronic excitotoxicity and increased calcium influx, as seen in TBI, trigger downstream signalling pathways that can lead to aberrant tau phosphorylation and cell death [363–365]. Both depolarization of neurons and damage to the integrity of the cell membrane following TBI results in the uncontrolled release of glutamate in the synaptic cleft, leading to an increase in post-synaptic calcium influx into the cytosol [361, 362, 366, 367]. Glutamate promotes calcium membrane permeability and influx by binding to ionotropic N-methyl D-aspartate (NMDA) and 2-amino-3–5-methyl-4-isoxazoleproprionic acid (AMPA) receptors [359, 368]. TBI can enhance aberrant glutamatergic signaling by upregulating the expression of NMDARs and AMPARs through cytokine signaling [369–371] and downregulating the levels of glutamate re-uptake transporters expressed on astrocytes [372]. This results in excess glutamate in the synaptic cleft, greater receptor activation and increased cytosolic calcium concentrations. Intriguingly, the pathophysiological effects of NMDAR activation are dependent on the specific expression of receptor subunits. Stimulation of NR2 A-containing NMDARs was associated with neuroprotection and JNK activation, whereas NR2B-containing NMDAR activation was associated with increased mitochondrial calcium accumulation and subsequent mitochondrial dysfunction [373]. Glutamate excitotoxicity induced by the administration of kainic acid in mice resulted in increased activation of CDK5 and JNK and downregulation of PP2 A, which was associated with increased tau phosphorylation, including CTE-relevant tau epitopes Ser202, Thr205, Thr217, and Ser396 [363]. Similar results were found in mouse hippocampal neurons, where glutamate excitotoxicity led to Thr231 tau phosphorylation, which was accompanied by CDK5 and GSK3β activation [364]. It has also been reported that tau itself can impact neuronal excitability and lead to subsequent excitotoxicity [374, 375]. Tau overexpression, which has been shown to reduce neuronal excitability, resulted in NMDAR-mediated cell death that was associated with the activation of ERK and calpain [376]. This effect may be in part mediated by tau phosphorylation as one study demonstrated that glutamate-induced calcium excitotoxicity was enhanced by the presence of pTyr18 tau [377].

Intracellular calcium levels are also regulated by efflux from intracellular endoplasmic reticulum stores via the ryanodine receptor (RyR) and inositol 1,4,5-trisphosphate (IP_3_) receptor (IP_3_R) [378]. The link between RyR and IP_3_R dysfunction and TBI is not fully understood, but alterations in receptor function have been implicated in other neurodegenerative diseases [379–383]. It has been proposed that RyRs can be activated by ROS [384] and calcium-induced calcium release in response to NMDAR activation [385]. Further, RyR-evoked calcium release is altered in a CCI model of TBI that was accompanied by increased p-tau [386]. Administration of Dantrolene, a RyR antagonist, was neuroprotective against hypoxia-induced brain injury, suggesting that targeting intracellular calcium mechanisms may be a potential therapeutic strategy [387]. IP_3_R is also a critical regulator of intracellular calcium stores, which is activated by IP_3_, the cleavage product of phosphatidylinositol bisphosphate by phospholipase C (PLC) [388]. In accordance, PLC and IP_3_ are elevated in TBI and stretch injury, respectively [389–391]. The expression level of IP_3_R may also be altered upon CNS trauma, as demonstrated by an increase in receptor expression following hypoxic injury [392].

Intracellular calcium overload can have many downstream effects, including the activation of tau kinases and deactivation of phosphatases, which in turn may lead to tau phosphorylation. Transcriptomic analysis of CTE post-mortem brain tissue revealed a downregulation in key genes associated with calcium signaling, suggesting calcium dysfunction plays a key role in the disease [157]. Membrane depolarization and subsequent increase in intracellular calcium in primary neurons led to increased PHF tau, which was associated with increased CDK5 and GSK3β activation [393]. Other in vitro studies have shown similar results concerning the effect of calcium signalling on tau phosphorylation including increases in pSer214, PHF and AT8 tau [394–396]. Intracellular calcium signalling also activates both caspases and calpains, proteolytic enzymes that have been linked to the pathophysiology of tau via kinase activation and cleavage of tau into neurotoxic fragments [397–400]. In fact, caspase-3 cleaved tau, which is present in various tauopathies, was shown to reduce endoplasmic reticulum calcium concentrations [401].

#### Mitochondrial dysfunction

Mitochondria play an essential role in the maintenance of metabolic homeostasis, contributing to healthy neuronal functioning. Alterations in calcium levels, as a result of neuronal excitotoxicity, have been shown to directly impact mitochondrial signalling and function, which can indirectly contribute to tau phosphorylation [402, 403]. As a result of increased intracellular calcium levels, the mitochondria attempt to regulate cytosolic calcium levels by increasing uptake through the mitochondrial uniporter (MCU) [404]. While this is initially a physiologically protective mechanism to maintain calcium homeostasis in response to intracellular calcium overload, excess mitochondrial calcium levels can induce mitochondrial dysfunction and activate a pathological signalling cascade. Mitochondrial dysfunction is critical in inducing oxidative stress through the production of excess free radicals. The accumulation of free radicals results in increased mitochondrial membrane permeability, allowing for their release into the cytosol [405]. Additionally, in states of dysfunction or disease, the mitochondria release cytochrome-c, a signalling molecule that can trigger programmed apoptotic cell death [406], which is a neuropathological hallmark of CTE.

Mitochondrial dysfunction can indirectly increase tau phosphorylation by the induction of oxidative stress and the release of free radicals, which can activate tau kinases (discussed further in the next section). In P301S transgenic mice, mitochondrial dysfunction preceded tau pathology, suggesting it may be an early indicator of tau pathogenesis [407]. The major physiological role of mitochondria is the production of ATP via oxidative phosphorylation, and both in vitro and in vivo studies have linked impaired oxidative phosphorylation to tau dysmetabolism. For example, administration of the ATP-depleting neurotoxin annonacin in primary neurons resulted in tau redistribution from the axons and subsequent cell death [408]. This was then reversed with the restoration of mitochondrial NADH oxidation in complex-I. In line with this, inhibition of complex-I in vivo resulted in an increase in AT8 p-tau in the striatum that was also co-labelled with thioflavin-S, indicating fibril formation [409].

In contrast, it has been shown that pathological tau itself has a significant impact on mitochondrial function and localization [410, 411], likely propagating feedforward mechanisms of tau pathogenesis. Accordingly, iPSCs harbouring pathogenic FTD-causing tau mutations have an altered interactome with mitochondrial proteins, including decreased binding to subunits of the electron transport chain [412]. In turn, this was associated with decreased mitochondrial function, including altered bioenergetics and ATP production. This is consistent with other studies that have demonstrated pathological tau is associated with impaired mitochondrial ATP synthesis [413–415]. Reduced ATP production results in the failure of the cell to accommodate the increased energy demand caused by TBI and secondary injury responses, exacerbating tau pathophysiology.

The maintenance of healthy mitochondrial function is a tightly regulated process that requires consistent organelle turnover through mitochondrial fission and fusion. This process is especially critical in injury and disease to control the turnover of damaged mitochondria without affecting mitochondrial biogenesis. Mitochondrial fission is mediated in part by the GTPase Dynamin-related protein 1 (Drp1) [416, 417]. Phosphorylated tau has been demonstrated to directly interact with Drp1, leading to increased GTPase activity and increased mitochondrial fragmentation [418]. Accordingly, partial knockdown of Drp1 in P301L mutant mice reduced pThr181 and pSer202/Thr205 tau, increased mitochondrial biogenesis and reduced dysfunction [419].

Additionally, a critical component in mitochondria biogenesis and function is the proper axonal transport of mitochondria along the microtubules to its intended cellular compartment. Given that pathological tau disrupts axonal transport, the presence of phosphorylated tau, as observed in CTE, may alter mitochondria transport and induce dysfunction. The effect of tau, including AT8 p-tau, on mitochondrial transport has been demonstrated in multiple studies [80, 420, 421]. Conversely, mitochondrial mislocalization in *Drosophila* led to an accumulation of pSer262 tau [422].

TBI has been shown to cause mitochondrial dysfunction in both humans and animal models [402, 423, 424]. Using magnetic resonance spectroscopy, patients with diffuse TBI had reduced levels of N-acetylaspartate (NAA), a metabolite that is synthesized in the mitochondria and has been used as a biomarker for mitochondrial impairment [424]. Further, the reduction was greater in TBI patients who had worse outcomes and poorer recovery at 40 days post-injury. Similarly, a controlled cortical impact model of TBI in rodents resulted in impaired mitochondrial electron transport chain function, which persisted for 14 days post-injury [423] suggesting TBI can induce chronic mitochondrial dysfunction. Evidence for mitochondrial impairment (beyond leading to the induction of oxidative stress) in CTE patients is scant but has been well-defined in other tauopathies including Alzheimer’s disease and FTD [406, 410, 425].

#### Oxidative stress

Oxidative stress is one of the most well-characterized components of the pathophysiology of TBI and neurodegenerative disease, which is caused by the imbalance in the production and neutralization of free radicals, including reactive nitrogen species (RNS) and ROS [426, 427]. Impairments in mitochondrial oxidative phosphorylation lead to the production of ROS and RNS, which are then released into the cytosol following mitochondrial membrane permeabilization and can trigger neurodegenerative signaling mechanisms leading to tau phosphorylation, neuroinflammation, and cell death [426–428]. The most common ROS and RNS implicated in TBI and CTE are superoxide, hydroxide, hydrogen peroxide, peroxynitrite, and nitric oxide [429–431]. Elevation of oxidative stress by hypoxia exposure prior to TBI in mice was associated with more severe outcomes and decreased metabolites, including NAA, compared to TBI only [432]. Similarly, it has been shown that molecular markers of oxidative stress also increased following TBI in humans [433, 434].

Upon oxidative stress, ROS and RNS can act upon various cellular pathways and disrupt cellular homeostasis. In doing so, oxidative stress has been most notably associated with apoptosis and neuronal damage through lipid peroxidation, proteolysis, and DNA damage, further exacerbating the neurodegenerative process [426, 428, 435]. In addition, oxidative stress can also trigger the activation of various kinases, and multiple studies have demonstrated that induction of oxidative stress in vitro leads to increased tau phosphorylation through this avenue [436–439]. The impact of oxidative stress on tau phosphorylation was associated with decreased PP2 A activity and increased activity of tau-related MAPKs, JNK, p38, and ERK [436, 440]. Other known tau-kinases, including GSK3β, are also increased by oxidative stress in vitro and are associated with increased tau phosphorylation [441, 442]. Biochemical analysis of P301L mice showed a progressive increase in ROS over time, which correlated with age-dependent increases in tau pathology [413]. Oxidative stress has also been shown to downregulate Pin1, which has implications for the genesis of pathological *cis* p-tau [443].

Under physiological conditions, increased production of ROS and RNS is compensated by the presence of antioxidants, including superoxide dismutase (SOD) and glutathione (GSH), which scavenge and reduce free radicals to protect the cell from oxidative stress [444]. However, following TBI and in neurodegenerative diseases, antioxidant mechanisms are impaired and unable to scavenge free radicals, thus increasing oxidative stress-induced tau phosphorylation [445]. Reduced antioxidant expression and activity have been linked to neurodegenerative disease, most notably in relation to Alzheimer’s disease [426]. With respect to TBI, one study demonstrated that a single moderate CCI in rats resulted in decreased levels of GSH, SOD, and catalase in the hippocampus within the first 12–72 h [446]. The decrease in antioxidants was associated with increased levels of ROS and subsequent synaptic dysfunction. Another study utilizing a blast model of TBI in mice showed an increase in SOD within 24 h post-injury [230]. Taken together, this demonstrates the complexity of the secondary injury response in TBI, such that different injury models can lead to opposing cellular alterations.

Induction of chronic oxidative stress in human neuroblastoma cells by inhibition of the antioxidant GSH increased hyperphosphorylated PHF tau [436]. In line with this, knockout of the mitochondrial SOD2 increased p-tau at residues Thr205, Thr231, Ser396, and Ser404 [447]. This effect was further exacerbated when SOD2 was knocked out in a transgenic Alzheimer’s disease mouse model overexpressing amyloid precursor protein (APP). Furthermore, antioxidant treatment attenuated the levels of p-tau in the SOD2 knockout mice, validating that tau phosphorylation is driven by oxidative stress and can be reduced with antioxidant therapy [447].

## Conclusion

CTE is a devastating neurodegenerative disease characterized by widespread neuropathological alterations and pronounced clinical symptoms. To date, CTE can only be diagnosed post-mortem by the presence of distinct phosphorylated tau lesions and no effective therapeutic treatments exist. While exposure to repeated head impacts is the primary risk factor for CTE, the exact mechanisms by which TBI leads to CTE are not fully understood. Although multiple studies have linked TBI and tau pathology, how TBI (or repetitive TBI in the case of CTE) induces tau pathology is unclear given the complex nature of the cellular mechanisms associated with TBI. The cellular environment elicited post-TBI is unique due to the rapid nature in which multiple converging pathways are activated. As outlined, many of the signaling cascades activated post-TBI can influence various cell types and multiple molecular pathways, inducing a feed-forward cycle that results in chronic activation and promotes tau pathology. Better understanding the role that neuroinflammation, excitotoxicity, mitochondrial dysfunction, and oxidative stress play in driving tau phosphorylation in concert with each other is critical for beginning to discover better biomarkers and drug candidates for CTE.

Despite the recent advancements in tau, TBI, and CTE, there remain many important considerations that require further investigation. While TBI has been linked to tau dysfunction, it is still unknown whether the presence of phosphorylated tau aggregates is exclusively responsible for the clinical manifestations of CTE. As discussed, CTE can present with co-pathologies, including TDP-43 and Aβ, and some cases are comorbidly diagnosed with disorders such as ALS or Parkinson’s disease [448]. At a different level, tau is subject to many different types of post-translational modifications in addition to phosphorylation, which may have critical implications in the development of tau pathology in TBI and CTE. In this review we have focused solely on tau phosphorylation, but other studies have demonstrated the importance of other post-translational modifications in driving tau aggregation, such as acetylation [101]. Different post-translational modifications can have differing effects on the aggregation, spreading and seeding of tau and may be a critical factor for strain variability between tauopathies.

Understanding the exact relationship between TBI and CTE is also a critical gap in knowledge that needs to be addressed. Unanswered questions surrounding the threshold of TBI or head impacts required to trigger CTE, and the link between the severity of head impacts and the severity of CTE remain. This becomes difficult to address given the variability in head impacts, such that two equivalent TBIs may have drastically different effects on differing individuals [449, 450]. Moreover, symptoms do not always indicate severity. At a research level, modelling TBI has always carried limitations, but newer approaches utilizing closed-head models and repetitive injury paradigms better replicate the physical and physiological environment that occurs in humans. From the tau perspective, selecting the appropriate animal model is essential for testing the appropriate hypothesis.

As the search for biomarkers and treatments for CTE continues, tau remains one of the most widely investigated targets given it is the pathognomonic requirement. The phosphorylation of tau is essential to the aggregation and formation of NFTs, and understanding the role that neuroinflammation, excitotoxicity, mitochondrial dysfunction, and oxidative stress play in promoting it can unveil potential targets to prevent tau phosphorylation and mitigate disease. Identification of imaging and fluid biomarkers that target tau and p-tau species has recently garnered attention but currently lacks specificity in distinguishing between certain tauopathies [34, 35, 451]. Similarly, various attempts to target tau as a therapeutic have been attempted but have yet to succeed beyond clinical trials [452, 453]. One approach may be to begin tailoring treatments and biomarkers to specific tauopathies. Given the recent advances in our understanding of the unique conformational variability between different tauopathies including CTE [250, 251], it is evermore crucial to examine tau pathobiology in disease-specific contexts. The differences between tau conformers or strains are crucial for the heterogeneity in the development, progression, and clinical presentation of different tauopathies. Combining our knowledge of the physiological, structural, pathological and clinical aspects of CTE is critical for the advancement of effective diagnostics and therapeutics.

## Data Availability

Not applicable.

## Abbreviations

Aβ: Amyloid beta
ALS: Amyotrophic lateral sclerosis
ALS-PDC: Amyotrophic lateral sclerosis/parkinsonism-dementia complex
AMPA: 2-Amino-3–5-methyl-4-isoxazoleproprionic acid
APOE4: Apolipoprotein E ε4
APP: Amyloid precursor protein
CAMKII: Calcium-calmodulin-dependent protein kinase II
CBD: Corticobasal degeneration
CCI: Controlled cortical impact
CDK5: Cyclin-dependent kinase 5
CHI: Closed head injury
CNS: Central nervous system
CSF: Cerebrospinal fluid
CTE: Chronic traumatic encephalopathy
DAM: Disease-associated microglia
DAMP: Disease-associated molecular pattern
Drp1: Dynamin-related protein 1
ERK: Extracellular signal-regulated kinase
FPI: Fluid percussion injury
FTD: Frontotemporal dementia
FTDP-17: Frontotemporal dementia with parkinsonism linked to chromosome 17
FTLD: Frontotemporal lobar degeneration
GSH: Glutathione
GSK3β: Glycogen synthase kinase-3 beta
HMGB1: High mobility group box protein 1
IFN: Interferon
IL: Interleukin
IP_3_R: Inositol 1,4,5-trisphosphate receptor
JNK: C-Jun N-terminal kinase
LPS: Lipopolysaccharide
MAC: Membrane attack complex
MAPK: Mitogen-activated protein kinase
MAPT: Microtubule-associated protein tau
MCU: Mitochondrial calcium uniporter
MKK: MAPK kinase
MTBD: Microtubule-binding domain
Myd88: Myeloid differentiation primary response 88
NAA: N-acetyl aspartate
NFT: Neurofibrillary tangle
NF-κB: Nuclear factor-kappa B
NLR: Nod-like receptor
NMDA: N-methyl D-aspartate
PAD: Phosphatase activating domain
PHF: Paired helical filament
Pin1: Peptidyl-prolyl cis/trans isomerase NIMA-interacting 1
PKA: CAMP-dependent protein kinase
PLC: Phospholipase c
PP1/2a: Protein phosphatase 1/2a
PSP: Progressive supranuclear palsy
RNS: Reactive nitrogen species
ROS: Reactive oxygen species
RyR: Ryanodine receptor
SOD: Superoxide dismutase
STAT: Signal transducer and activator of transcription
TBI: Traumatic brain injury
TDP-43: TAR DNA-binding protein 43
TES: Traumatic encephalopathy syndrome
TGFβ: Transforming growth factor beta
TLR: Toll-like receptor
TMEM106B: Transmembrane protein 106B
TNF-α: Tumor necrosis factor-alpha
TOC1: Tau Oligomeric Complex 1
TREM2: Triggering receptor expressed on myeloid cells 2
